# Efficacy of a Cognitive Behavioral Therapy–Based Online Self-Help Group for Depression and Suicide Ideation: Randomized Controlled Trial

**DOI:** 10.2196/76028

**Published:** 2026-07-03

**Authors:** Minkyung Yim, Haeun Kim, Soo-Eun Lee, Eunjin Jo, Ji-Won Hur

**Affiliations:** 1 School of Psychology, Korea University, #115, College of Law Annex (Old Law Bldg), 145 Anam-ro, Seoul, 02841, Republic of Korea, 82 2-3290-2063; 2 Department of Psychiatry, Sungkyunkwan University College of Medicine, Samsung Medical Center, Seoul, Republic of Korea; 3 Department of Psychology, Virginia Commonwealth University, Richmond, Virginia, United States; 4 Department of Psychiatry, Kangbuk Samsung Hospital, Seoul, Republic of Korea

**Keywords:** depression, self-help group, suicidal ideation, randomized controlled trials, cognitive behavioral therapy

## Abstract

**Background:**

Despite the high prevalence of depressive disorders, access to effective treatment remains limited due to financial, geographic, and social barriers. Online self-help groups offer a promising and scalable form of peer-based support beyond traditional clinical settings. Integrating cognitive behavioral therapy (CBT) techniques such as cognitive restructuring and behavioral activation into self-help groups may enhance their effectiveness.

**Objective:**

This study evaluated the efficacy of a cognitive behavioral therapy–based online self-help group (COS) that integrates structured CBT techniques with peer-led group support as a low-intensity intervention for individuals with depressive symptoms. A randomized controlled trial (RCT) comparing COS with a CBT-based mobile application was conducted. Additionally, a separately recruited waitlist control group was included as a supplementary comparison condition.

**Methods:**

Participants were recruited online. After eligibility screening via a structured clinical interview, participants were randomly assigned to a COS group (n=79) or a CBT-based mobile application group (n=39). An additional waitlist control group (n=48) was recruited separately during the second phase of the study. The COS intervention involved 7 videoconferencing sessions that incorporated peer-led group discussions, sharing lived experiences, and core CBT techniques such as cognitive restructuring. The primary outcome measure was depressive symptoms, assessed using the Beck Depression Inventory-II, and the secondary outcome was suicidal ideation, estimated using the Beck Scale for Suicide Ideation, measured at baseline, postintervention, and 3-month follow-up. Linear mixed models were used to evaluate group × time interaction effects. Reliable change indices were also calculated to assess clinical significance. All statistical tests were 2-tailed.

**Results:**

Among participants assigned to the COS group, 61% (48/79) completed all 7 sessions, and 84% (66/79) attended 5 or more sessions. A significant time × group interaction was observed for depressive symptoms (*F*_4,288.47_=7.23, *P*<.001). The COS group exhibited a substantial reduction in depressive symptoms from baseline to postintervention (*t*_285.76_=10.77, two-tailed; *P*<.001), with a large within-group effect size (*d*=1.38); this improvement was maintained at the 3-month follow-up. Suicidal ideation also significantly decreased in the COS group (*t*_277.11_=4.55, two-tailed; *P*<.001), with sustained effects at follow-up. Clinically meaningful improvement in depressive symptoms, as defined by the reliable change index, was observed in 75% (56/75) of COS participants. While both the COS and app-based CBT groups achieved comparable reductions in depressive symptoms, only the COS group demonstrated a significant reduction in suicidal ideation.

**Conclusions:**

This RCT provides evidence that a structured, CBT-informed online self-help group can reduce depressive symptoms and suicidal ideation. The COS program offers a scalable, accessible alternative to traditional therapy, particularly in settings with limited access to mental health professionals.

## Introduction

Depressive disorders have far-reaching public health consequences, contributing to work absenteeism, increased economic burden, elevated suicide risk, and higher all-cause mortality [1-3]. The duration of untreated depression is a critical determinant of clinical outcomes; shorter durations are associated with faster remission and better treatment response [4]. Thus, timely access to effective intervention is essential for mitigating the course and consequences of depressive illness [5,6].

Despite this urgency, approximately 30% of individuals diagnosed with depression receive no treatment at all [7]. Common barriers include financial hardship, geographic inaccessibility, stigma, and a shortage of qualified mental health professionals [8,9]. These systemic barriers underscore the need for alternative models of care that are both accessible and scalable. Digital health technologies, including videoconferencing and mobile applications, have expanded access to mental health care by providing flexible, cost-effective, and evidence-based interventions for underserved populations [10-13]. Among these, online self-help groups are promising platforms for delivering support outside traditional clinical settings [14].

Traditional self-help groups are typically peer-led and rooted in shared lived experiences [15], fostering mutual support among individuals facing common challenges [14,16,17]. Participation in these groups has been associated with reduced depressive symptoms [18,19], improvements in quality of life [20], decreased feelings of loneliness [21], and overall reductions in mental health problems [22,23]. These benefits, supported by meta-analyses and systematic reviews [18,20,22,24], underscore the potential value of self-help groups as scalable, low-barrier interventions.

However, the utility of traditional in-person self-help groups is often curtailed by significant structural impediments. Community-based local groups depend on physical proximity, regular attendance, and a sufficient number of members to maintain operations, which often serve as barriers to entry and sustainability [25]. Individuals residing in rural areas, or those burdened by mobility limitations, physical illness, caregiving responsibilities, or irregular working hours, often find in-person attendance untenable [25-27]. Furthermore, in smaller communities, concerns regarding anonymity or privacy may deter participation, while low population density may preclude the formation of groups entirely. These persistent barriers underscore the urgent need for delivery formats capable of extending the reach of self-help interventions beyond local constraints.

The application of digital health technologies, particularly real-time videoconferencing, offers a robust solution to these challenges. By enabling participation irrespective of location, reducing the burden of travel, and facilitating flexible scheduling, these technologies effectively mitigate traditional barriers while enhancing anonymity [28,29]. Crucially, synchronous online formats preserve key therapeutic elements of self-help groups: mutual support and real-time emotional exchange among individuals with lived experiences. Consequently, online self-help groups represent a vital mobile health innovation, leveraging the technology to connect participants nationwide and expanding access to structured psychological support where traditional models fall short.

The term “online self-help group” encompasses a diverse range of formats, ranging from large, asynchronous text-based discussion forums to synchronous, small group-based mutual support sessions. This study focuses specifically on the latter—synchronous, videoconferencing-based groups designed to foster real-time interaction and support. Despite the promise of this format, there is a notable gap in the literature [30]: to date, few randomized controlled trials (RCTs) have rigorously evaluated the clinical efficacy of synchronous online self-help groups in treating depression. Moreover, a key limitation of typical self-help groups is their lack of structured therapeutic content, resulting in wide variability in group facilitation, composition, and outcomes [31]. This inconsistency poses challenges to their reliability and scalability as interventions for individuals with clinically significant depressive symptoms. In this study, we developed a semistructured manual for a cognitive behavioral therapy–based online group self-help program (COS). We then examined its efficacy among individuals experiencing depressive symptoms through an RCT. The COS program integrated core cognitive behavioral therapy (CBT) components, including cognitive restructuring and coping skills, to enhance therapeutic consistency and effectiveness within a peer-led group format. This hybrid model builds on prior work, such as SMART Recovery [32], showing that structured CBT elements can improve psychological outcomes in mutual aid settings.

A noninteractive, CBT-based mobile application served as the active comparator. While this application has demonstrated efficacy in reducing depressive symptoms through interventions targeting dysfunctional thoughts [33], it is designed for individual use and does not offer the social support and accountability that characterize group-based interventions such as the COS program. In addition, a separately recruited waitlist control group was included to account for potential symptom improvement attributable to the natural course of depression.

Consequently, we hypothesized that the COS program would be at least as efficacious as the CBT-based mobile application intervention for general CBT-based outcomes, such as depressive symptoms, stress, and dysfunctional attitudes, and that the peer-based format of COS might offer additional benefits for outcomes involving emotional or interpersonal processes. Furthermore, we expected that a higher proportion of individuals in the COS group would demonstrate clinically meaningful improvement.

To our knowledge, few RCTs have evaluated online self-help groups for depression, and prior studies have typically examined only unstructured peer-support interventions. This study contributes to this literature by examining a semistructured, CBT-informed online self-help group and by including an active digital CBT comparator. We anticipate that the findings will highlight the potential of online self-help groups as a scalable, low-barrier intervention that can help overcome the logistical and geographic challenges often related to accessing traditional, evidence-based practices.

## Methods

### Study Design

This study primarily used a 2-arm RCT in which participants were randomly assigned to either the COS or the cognitive behavioral therapy–based mobile application (APP) group at a 2:1 ratio, using block randomization. Participants of the RCT were recruited between May and November 2021. Interventions were delivered between August and December 2021, and follow-up assessments were completed in March 2022.

To complement the primary RCT design, a nonrandomized waitlist control group was recruited during the second phase of the study from December 2021 to April 2022. This waitlist control group provided a baseline for the natural course of depression. While the active comparator (APP) has demonstrated efficacy in reducing depressive symptoms [33], relying exclusively on this digital intervention prevents a direct assessment of absolute efficacy relative to a no-treatment control. Therefore, following the completion of enrollment for the randomized arms, a waitlist control group was recruited to distinguish the intervention effect from spontaneous remission.

Participant enrollment proceeded in two distinct phases due to operational and timeline constraints. During the initial recruitment period, the research team prioritized the randomized comparison between the COS and APP groups, as this constituted the primary objective of the trial. To minimize potential confounding, identical inclusion and exclusion criteria were applied across phases, and sociodemographic characteristics were monitored rigorously.

### Recruitment and Screening Procedures

Participants for the RCT were recruited between May and November 2021 through online advertisements disseminated via social media platforms (eg, X and Instagram) and university-based online community platforms in Korea. Interested individuals were invited to contact the research team via direct message or email to receive detailed study information and to schedule an initial telephone screening.

Inclusion criteria were as follows: (1) aged between 19 and 55 years, (2) diagnosed with any depressive disorder based on the Structured Clinical Interview for DSM-5 – Clinician Version (SCID-5-CV) or having a PHQ-9 (Patient Health Questionnaire-9) score of ≥5 indicating mild depression [34], (3) fluent in Korean, and (4) able to use a real-time videoconferencing program. Participants undergoing pharmacotherapy were eligible only if their dosage had been stable for at least 1 month.

Exclusion criteria included current psychotic symptoms (ie, hallucinations and delusions), concurrent psychological interventions, or high suicide risk. Individuals with high suicide risk, defined as those reporting suicide ideation with planning during the SCID-5-CV, were excluded and referred to appropriate services. No participants were excluded on this basis.

Recruitment followed a 2-step screening procedure. Interested individuals first completed a brief telephone screening that assessed medication stability, current psychological intervention, and commitment to the study schedule. Eligible participants received a brief explanation of the study after eligibility screening (SCID-5-CV) and provided informed consent via an online signature. Of 186 individuals initially screened, 141 underwent online SCID-5-CV interviews and initial assessments. SCID-5-CV interviews were conducted by graduate students in clinical and counseling psychology or licensed clinical psychologists, all of whom received more than 2 weeks of SCID-5-CV administration training. A total of 127 participants were randomized to the COS or APP group.

For the recruitment and screening of the waitlist control group, the same inclusion and exclusion criteria used for the RCT were applied to the waitlist control, except that the requirement for real-time videoconferencing ability was waived. During this phase, 74 individuals were screened, 55 completed eligibility procedures, and 48 were enrolled. The study adhered to the CONSORT (Consolidated Standards of Reporting Trials) 2010 guidelines [35], and the CONSORT checklist is provided as Checklist 1.

### Sample Size Calculation

The sample size was determined based on power estimates and feasibility considerations. We adopted a statistical power of 0.80 and a significance level of α=.05. The assumed effect size was set to *f*=0.25, a conservative estimate informed by prior meta-analyses of digital psychological interventions and peer support–based approaches, both of which generally report small-to-moderate improvements in depressive symptoms [36,37]. Using *f*=0.25 for detecting a group × time interaction in a repeated-measures design, a total of approximately 66 participants were required. Given the 2:1 randomization ratio between the COS and APP groups, we aimed to recruit at least 99 participants to ensure adequate power.

### Randomization

Block randomization was performed using a computer-generated list, with a fixed block size of 3. Participants were randomly assigned to the COS or APP group at a 2:1 ratio.

### Interventions

#### The COS Program

**Figure 1.**
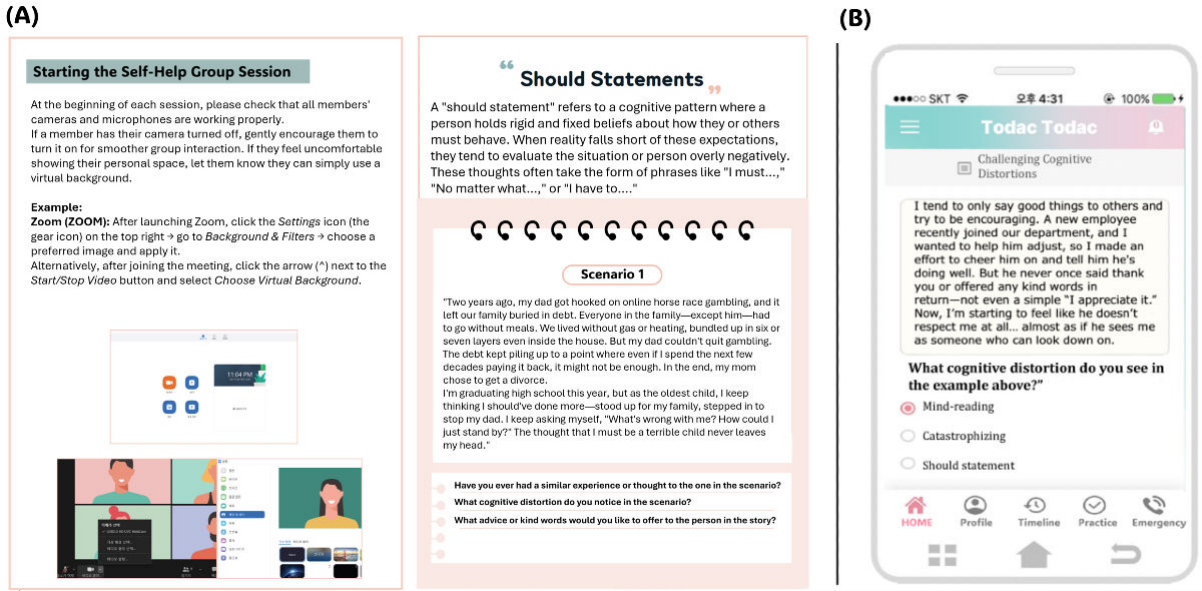
(A) Sample page from the participant workbook provided in the COS program. (B) Screenshot of the cognitive distortion exercise featured in the cognitive behavioral therapy–based mobile application. These materials were originally developed in Korean and translated into English. COS: cognitive behavioral therapy–based online self-help group.

The COS program is a semistructured, 7-session, CBT-based online self-help group protocol designed to alleviate depressive symptoms. The COS manual and workbooks were developed and reviewed by licensed clinical psychologists to ensure fidelity to the self-help group format and CBT principles and accuracy of therapeutic content. Figure 1A shows a sample page from the participant workbook provided in the COS program.

Each group typically consisted of 4‐6 participants and 1 facilitator, with each session lasting approximately 50 minutes. Sessions were held twice weekly via videoconferencing platforms such as Zoom. A face-to-face pilot version of the COS program was conducted in 2020 to refine content and structure prior to web-based adaptation [38]. The first session of COS focused on rapport-building, sharing lived experiences with depression, and psychoeducation. Subsequent sessions (2-7) focused on introducing various cognitive distortions (eg, catastrophizing, mind reading, overgeneralization, “should” statements, tunnel vision, and black-and-white thinking). In each session, participants read a series of scenarios written in the manual of the COS program, reflecting on each cognitive distortion and suggesting possible alternatives for the protagonist. After reading the given scenario, the participants were encouraged to share personal experiences related to these negative and distressing emotions related to each cognitive distortion. These activities were designed to provide participants insight into their negative automatic thoughts and emotional support from others with similar experiences.

Although the COS program was fundamentally designed to permit participants to assume the facilitator role, a designated facilitator who was either a graduate psychology student or a licensed clinical psychologist was assigned in this study. This was implemented to ensure strict adherence to protocols that monitor participant safety, in accordance with research ethical standards. To facilitate the intended peer-led model, the program includes a publicly available participant manual and a step-by-step facilitator guidebook, alongside a brief introductory video provided through the Korean National Center for Mental Health. Crucially, substantive discussions, including the sharing of lived experiences and the restructuring of cognitive distortions, remained participant-led. All facilitators received 30 to 40 minutes of training on COS procedures before implementation. Their role was strictly limited to logistical management, such as opening the videoconferencing sessions, introducing session activities, and timekeeping, alongside the monitoring of participant safety. Facilitators were trained to monitor participant safety and to notify the research team immediately if a participant reported active suicidal thoughts. In such cases, they were instructed to administer the P4 screener [39] to assess suicide risk and to contact the research team for a follow-up safety evaluation. No instances of active suicidal ideation were reported during the study.

#### The CBT-Based Mobile Application

We used a CBT-based mobile application as an active comparator. The efficacy of the app was confirmed in an RCT [33]. Similar to the COS program, the app presented short stories illustrating frustrating situations that highlighted various cognitive distortions, followed by quizzes designed to identify these distortions and reduce related negative emotions (Figure 1B). Participants in the APP group were instructed to use the app for approximately 270 to 360 minutes (5 minutes per session × 3 sessions daily × 24 days), ensuring comparable total usage time to the COS program. Weekly reminders were sent to encourage adherence to the app usage schedule. Participants were considered to have completed the APP program if they used the app for at least 18 days (270 minutes), completing 3 or more sessions per day.

### Outcome Measures

The primary outcome was depressive symptoms, assessed using the Beck Depression Inventory-II (BDI-II), a widely used measure of depression [40]. Scores from 0 to 13 indicate no or minimal depressive symptoms, 14 to 19 mild, 20 to 28 moderate depression, and 29 to 63 severe depressive symptoms.

The secondary outcome was suicidal ideation, measured using the Beck Scale for Suicide Ideation (BSS). The BSS consists of 21 items that assess the severity of suicidal symptoms [41].

Exploratory outcomes included cognitive and psychosocial variables. Dysfunctional attitudes, a predisposing cognitive structure underlying depression, were measured using the Dysfunctional Attitude Scale (Form A) Revised (DAS-17) [42,43]. Daily life stressors were measured using the Perceived Stress Scale (PSS) [44]. Loneliness and perceptions of social support were assessed using the UCLA Loneliness Scale version 3 (UCLA-LS) [45,46] and the Perceived Available Support (PAS) scale of the Berlin Social Support Scale (BSSS) [47], respectively. Protective factors against suicide risk were assessed using the Reasons for Living Inventory for Young Adults (RFL-YA) [48]. Finally, general self-efficacy was measured using the General Self-Efficacy Scale (GSES) [49], given its known association with depression. All outcome measures were administered via Qualtrics as online self-report questionnaires.

### Statistical Analysis

An intent-to-treat analysis was conducted to compare the immediate and 3-month efficacy of the programs using a linear mixed model (LMM). Prior to the main analysis, the appropriateness of the LMM for the repeated-measures design was confirmed by evaluating within-participant dependence using unconditional mixed-effects models. Intraclass correlation coefficients (ICCs) were calculated for this preliminary assessment. A significant (*P*<.05) interaction between time and condition in the hypothesized directions indicated that one group showed greater improvement.

Prior to the main analysis, we examined the missingness mechanism for the primary outcome, BDI-II. Little’s missing completely at random (MCAR) test was insignificant (*χ*²_3_=0.96, *P*=.62), and baseline BDI-II scores did not predict missingness (*P*=.24), supporting a missing at random (MAR) mechanism. Missing data were not imputed because the mixed-model approach provided equal or better power without imputing missing data for the intent-to-treat analysis under MAR assumptions [50]. To test the efficacy of the intervention, we evaluated the differences in scores between groups at each time point (T_1_, T_2_, and T_3_). In the LMM, all the analyses included concurrent medication and social contact frequency as covariates. For post hoc analyses, both uncorrected *P* values and Holm-Bonferroni corrected *P* values (*P*_holm_) were reported. Effect sizes were calculated using Cohen *d*. Within-group effect sizes for within-group changes were adjusted for correlations between repeated measurements to account for the dependency between time points [51-53].

To determine the statistical significance of the changes in the primary outcome measures between baseline and postintervention, we calculated a reliable change index (RCI) [54,55]. The RCI was computed using the RCI calculator [56]. A significant RCI indicates that the observed change in an individual participant’s score over time is statistically meaningful. We used chi-square analysis to compare the proportions of individuals in each group demonstrating meaningful improvements or deterioration. All statistical tests were 2-tailed.

### Ethical Considerations

The study protocol was reviewed and approved by the Institutional Review Board (IRB) of Korea University (KUIRB-2021-0157-06), and the trial was retrospectively registered with the Clinical Research Information Service (KCT0007673), a primary registry of the WHO International Clinical Trials Registry Platform.

All participants provided written informed consent after being informed about the study’s objectives, procedures, and potential risks and benefits. Participants were explicitly informed that their involvement was voluntary and that they retained the right to withdraw from the study at any time without penalty. To ensure privacy, all participant data were anonymized prior to analysis, with identifiable information removed. Data were stored securely on password-protected servers, with access restricted strictly to authorized research personnel. Compensation was provided for each component of the study, including the screening interview, intervention sessions, and assessments at baseline, postintervention, and the 3-month follow-up. Participants who completed all study components received a maximum total compensation of approximately KRW 115,000 (about US $78 as of December 5, 2025).

## Results

### Adherence and Baseline Sociodemographic and Clinical Characteristics

**Figure 2.**
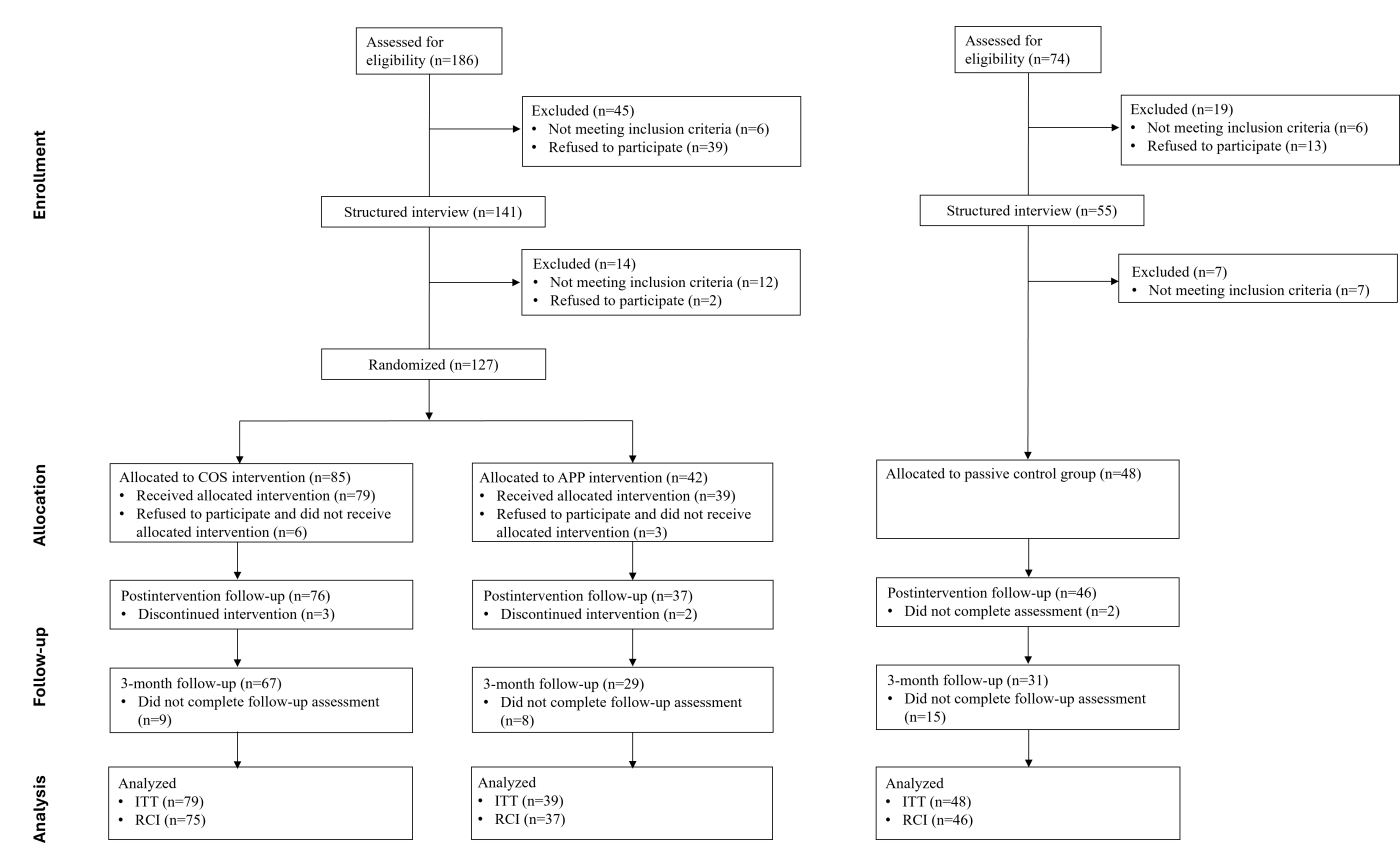
The CONSORT flow diagram of the randomized controlled trials (left) and waitlist control group (right). APP: cognitive behavioral therapy–based mobile application group; COS: cognitive behavioral therapy–based online self-help group; ITT: intent-to-treat analysis; RCI: reliable change index analysis.

Figure 2 presents the CONSORT participant flowchart. Of the 127 individuals randomized, 6 in the COS group and 3 in the APP group declined to participate prior to the start of the trial, resulting in 79 participants in the COS group and 39 in the APP group completing the baseline assessment (Table 1). In terms of adherence, 84% (n=66) of participants in the COS group attended 5 or more sessions of the program, and 82% (n=32) of those in the APP group used the app for at least 18 days.

**Table 1.** Baseline sociodemographic characteristics of the participants.

Characteristics	COS^a^ (n=79)	APP^b^ (n=39)	Waitlist control (n=48)	Chi-square (*df*) or *F* test (*df*)	*P* value
Age (years), mean (SD)	27.01 (6.05)	27.69 (6.82)	26.83 (7.47)	0.18 (2, 84.82)	.83
Sex, n (%)				1.32 (4)	.86
Female	71 (90)	34 (87)	44 (92)		
Male	7 (9)	4 (10)	4 (8)		
Prefer not to answer	1 (1)	1 (3)	0 (0)		
Sexual orientation, n (%)				3.07 (8)	.93
Heterosexual	55 (70)	29 (74)	38 (79)		
Gay or lesbian	1 (1)	1 (3)	1 (2)		
Bi- or pansexual	14 (18)	4 (10)	5 (10)		
Others	4 (5)	3 (8)	2 (4)		
Prefer not to answer	5 (6)	2 (5)	2 (4)		
Socioeconomic status, n (%)				11.27 (8)	.19
Lower	9 (11)	1 (3)	7 (15)		
Upper lower	16 (20)	5 (13)	12 (25)		
Middle	29 (37)	18 (46)	22 (46)		
Upper middle	24 (30)	14 (36)	6 (13)		
High	1 (1)	1 (3)	1 (2)		
Educational attainment, n (%)				2.25 (4)	.69
High school	3 (4)	2 (5)	2 (4)		
College	64 (81)	27 (69)	38 (79)		
Graduate school	12 (15)	10 (26)	8 (17)		
BDI-II^c^, mean (SD)	27.03 (10.01)	26.72 (11.31)	26.48 (9.93)	0.05 (2, 87.31)	.96
BSS^d^, mean (SD)	9.78 (7.11)	8.31 (8.36)	7.54 (7.97)	1.36 (2, 85.44)	.26
Concurrent pharmacotherapy, n (%)				1.2 (2)	.55
Positive	19 (24)	7 (18)	8 (17)		
Negative	60 (76)	32 (82)	40 (83)		

a COS: cognitive behavioral therapy–based online self-help group.

b APP: cognitive behavioral therapy–based mobile application group.

c BDI-II: Beck Depression Inventory-II.

d BSS: Beck Scale for Suicide Ideation.

There were no significant differences across groups in age, sex, sexual orientation, socioeconomic status, or educational level (all *P*>.05). Individuals receiving concurrent pharmacotherapy accounted for 20% of all participants. Chi-square analysis revealed no significant differences between the groups (*P*=.55). Baseline depressive symptoms (as measured by the BDI-II) and suicidal ideation (as measured by the BSS) also did not differ significantly across groups (both *P*>.05).

### Primary Outcomes

Initial unconditional mixed-effects models indicated substantial within-subject clustering in depressive symptoms (ICC=0.59), supporting the use of LMM. Consistent with the study design, the primary comparison focused on the 2 randomized groups, COS and APP, while the waitlist group served as a nonrandomized comparison group to assess natural symptom fluctuation.

**Figure 3.**
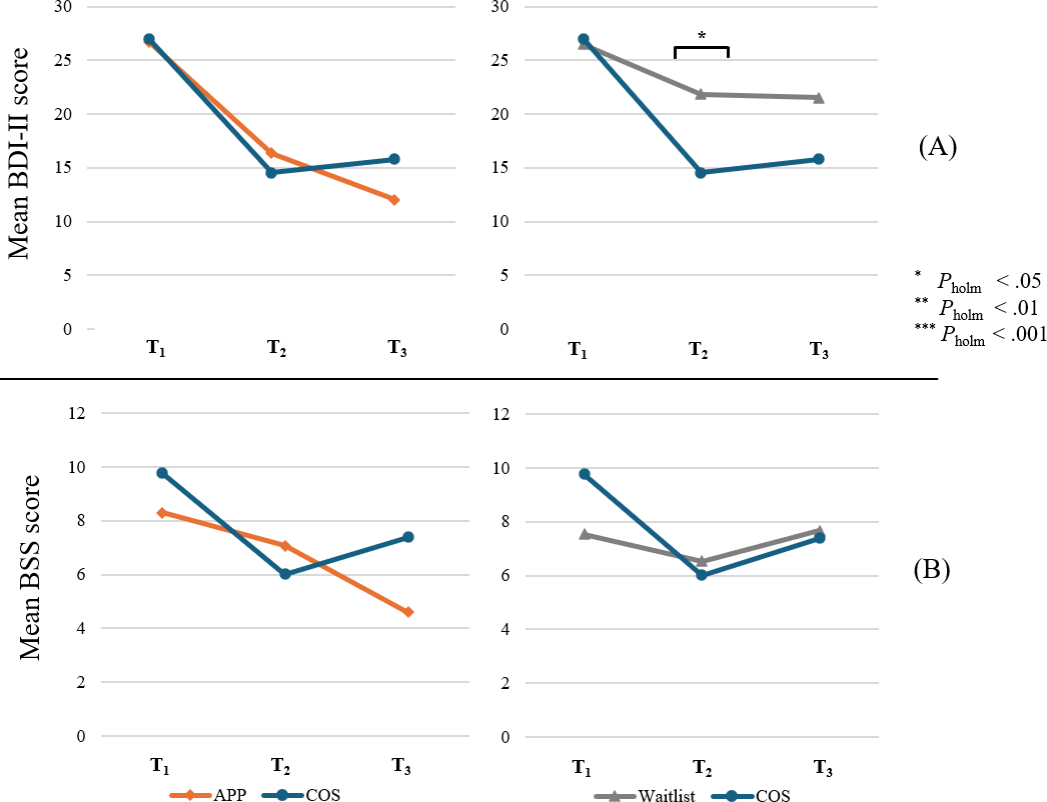
(A) Estimated mean BDI-II scores over time. (B) Estimated mean BSS scores over time. APP: cognitive behavioral therapy–based mobile application group; BDI-II: Beck Depression Inventory-II; BSS: Beck Scale for Suicide Ideation; COS: cognitive behavioral therapy–based online self-help group; *P*_holm_: Holm-Bonferroni corrected *P* values; Waitlist: waitlist control group.

The LMM analysis revealed significant main effects of time (*F*_2,289.99_=86.76, *P*<.001), group (*F*_2,163.68_=3.32, *P*=.04), and time × group interaction (*F*_4,288.47_=7.23, *P*<.001) for depressive symptoms, as measured by the BDI-II (Table 2). Figure 3A illustrates changes in depressive symptoms across T_1_, T_2_, and T_3_ for each group.

**Table 2.** Fixed effect omnibus test results for group and time.

Variables and effect	*F* test (*df*)	*P* value
BDI-II^a^		
Time	86.76 (2, 289.99)	<.001
Group	3.32 (2, 163.68)	.04
Time × group	7.23 (4, 288.47)	<.001
BSS^b^		
Time	7.66 (2, 277.57)	<.001
Group	0.23 (2, 161.83)	.80
Time × group	2.05 (4, 276.40)	.09
DAS-17^c^		
Time	21.2 (2, 288.82)	<.001
Group	1.67 (2, 164.81)	.19
Time × group	5.90 (4, 287.54)	<.001
PSS^d^		
Time	34.41 (2, 295.20)	<.001
Group	2.33 (2, 166.30)	.10
Time × group	2.72 (4, 293.49)	.03
GSES^e^		
Time	24.43 (2, 285.41)	<.001
Group	1.56 (2, 162.28)	.21
Time × group	4.21 (4, 284.24)	.003
UCLA-LS^f^		
Time	22.77 (2, 282.51)	<.001
Group	1.15 (2, 158.60)	.32
Time × group	3.21 (4, 281.24)	.01
PAS^g^		
Time	11.35 (2, 285.10)	<.001
Group	0.50 (2, 158.70)	.61
Time × group	0.49 (4, 283.46)	.75
RFL-YA^h^		
Time	14.26 (2, 281.49)	<.001
Group	1.19 (2, 159.51)	.31
Time × group	3.53 (4, 280.28)	.008

a BDI-II: Beck Depression Inventory-II.

b BSS: Beck Scale for Suicide Ideation.

c DAS-17: Dysfunctional Attitude Scale (Form A) Revised.

d PSS: Perceived Stress Scale.

e GSES: General Self-Efficacy Scale.

f UCLA-LS: UCLA Loneliness Scale version 3.

g PAS: Perceived Available Support.

h RFL-YA: Reasons for Living Inventory for Young Adults.

After each program (T_2_), both the COS and APP groups showed significant reductions in depression from baseline (COS: difference=11.84, *t*_285.76_=10.77, two-tailed, *P*_holm_<.001; APP: difference=10.26, *t*_283.29_=6.6, two-tailed, *P*_holm_<.001) (Table 3). Mean BDI-II scores decreased from the moderate range at baseline (COS: T_1_ mean 27.03, SD 10.01; APP: T_1_ mean 26.72, SD 11.31) to the mild range at postintervention (COS: T_2_ mean 14.57, SD 10.99; APP: T_2_ mean 16.35, SD 10.87) after the program in both groups (Table 4). Within-group comparisons indicated large effect sizes in both groups (*d*=1.38 for COS; *d*=0.93 for APP).

At the 3-month follow-up (T_3_), depressive symptoms in the COS group did not show a statistically significant change compared to T_2_ (difference=−0.72; *t*_283.76_=−0.62, two-tailed; *P*=.53). In the APP group, depressive symptoms decreased from T_2_ to T_3_ (difference=3.85; *t*_288.53_=2.25, two-tailed; *P*=.03), although this reduction did not remain significant after Holm-Bonferroni correction (*P*_holm_=.30). The difference between the COS and the APP group at T_3_ was not significant (difference=3.18; *t*_168.7_=1.43, two-tailed; *P*=.15), indicating comparable longer-term outcomes between the two randomized groups.

As secondary evidence, both the COS and the APP groups were compared with the waitlist control group. At T_2_, the COS group showed a significantly greater reduction in depressive symptoms compared with the waitlist control group (difference=−6.9; *t*_279.71_=−3.51, two-tailed; *P*<.001, *P*_holm_=.01, *d*=0.68), indicating a medium effect size. The APP group also showed lower depressive symptoms than the waitlist controls at T_2_, but this did not meet the Holm-Bonferroni correction (difference=−5.51; *t*_279.9_=−2.39, two-tailed; *P*=.02, *P*_holm_=.25).

At T_3_, the COS group showed lower depressive symptoms than the waitlist control group (difference=−5.43; *t*_330.55_=−2.56, two-tailed; *P*=.01), but this difference did not remain significant after Holm-Bonferroni correction (*P*_holm_=.17). The APP group showed significantly lower depressive symptoms than the waitlist control group at T_3_ (difference=−8.6; *t*_338.28_=−3.42, two-tailed; *P*<.001, *P*_holm_=.02).

**Table 3.** Time × group comparison for the primary and secondary measures.

	EMM^a^ (SE)	EMM^a^ (SE)	Difference	SE	*t* test (*df*)^b^	*P* value	*P^c^*_holm_
BDI-II^d^, comparison by time points							
T_1_ COS^e^ vs T_2_ COS	27.00 (1.26)	15.16 (1.29)	11.84	1.10	10.77 (285.76)	<.001	<.001
T_1_ COS vs T_3_ COS	27.00 (1.26)	15.88 (1.33)	11.12	1.15	9.71 (287.52)	<.001	<.001
T_2_ COS vs T_3_ COS	15.16 (1.29)	15.88 (1.33)	−0.72	1.15	−0.62 (283.76)	.53	≥.99
T_1_ APP^f^ vs T_2_ APP	26.81 (1.77)	16.55 (1.80)	10.26	1.56	6.6 (283.29)	<.001	<.001
T_1_ APP vs T_3_ APP	26.81 (1.77)	12.70 (1.92)	14.11	1.70	8.28 (292.08)	<.001	<.001
T_2_ APP vs T_3_ APP	16.55 (1.80)	12.70 (1.92)	3.85	1.71	2.25 (288.53)	.03	.30
T_1_ Waitlist^g^ vs T_2_ Waitlist	26.08 (1.64)	22.06 (1.66)	4.02	1.42	2.83 (284.55)	.005	.09
T_1_ Waitlist vs T_3_ Waitlist	26.08 (1.64)	21.30 (1.80)	4.78	1.58	3.02 (293.78)	.003	.06
T_2_ Waitlist vs T_3_ Waitlist	22.06 (1.66)	21.30 (1.80)	0.76	1.59	0.48 (288.52)	.63	≥.99
BDI-II, comparison by groups							
T_2_ COS vs T_2_ APP	15.16 (1.29)	16.55 (1.80)	−1.39	2.10	−0.66 (139.49)	.51	≥.99
T_2_ COS vs T_2_ Waitlist	15.16 (1.29)	22.06 (1.66)	−6.9	1.97	−3.51 (279.71)	<.001	.01
T_2_ APP vs T_2_ Waitlist	16.55 (1.80)	22.06 (1.66)	−5.51	2.31	−2.39 (279.9)	.02	.25
T_3_ COS vs T_3_ APP	15.88 (1.33)	12.70 (1.92)	3.18	2.24	1.43 (168.7)	.15	≥.99
T_3_ COS vs T_3_ Waitlist	15.88 (1.33)	21.30 (1.80)	−5.43	2.12	−2.56 (330.55)	.01	.17
T_3_ APP vs T_3_ Waitlist	12.70 (1.92)	21.30 (1.80)	−8.6	2.51	−3.42 (338.28)	<.001	.02
BSS^h^, comparison by time points							
T_1_ COS vs T_2_ COS	10.28 (0.95)	7.15 (0.96)	3.13	0.69	4.55 (277.11)	<.001	<.001
T_1_ COS vs T_3_ COS	10.28 (0.95)	8.22 (0.98)	2.06	0.71	2.91 (275.76)	.004	.14
T_2_ COS vs T_3_ COS	7.15 (0.96)	8.22 (0.98)	−1.07	0.71	−1.5 (274.21)	.13	≥.99
T_1_ APP vs T_2_ APP	9.08 (1.31)	7.88 (1.33)	1.2	0.94	1.27 (272.63)	.21	≥.99
T_1_ APP vs T_3_ APP	9.08 (1.31)	6.45 (1.41)	2.63	1.05	2.53 (278.73)	.01	.44
T_2_ APP vs T_3_ APP	7.88 (1.33)	6.45 (1.41)	1.43	1.05	1.36 (276.39)	.18	≥.99
T_1_ Waitlist vs T_2_ Waitlist	8.21 (1.22)	7.52 (1.23)	0.69	0.86	0.8 (273.74)	.43	≥.99
T_1_ Waitlist vs T_3_ Waitlist	8.21 (1.22)	7.68 (1.32)	0.53	0.99	0.54 (280.53)	.59	≥.99
T_2_ Waitlist vs T_3_ Waitlist	7.52 (1.23)	7.68 (1.32)	−0.16	0.99	−0.16 (276.51)	.87	≥.99
BSS, comparison by groups							
T_2_ COS vs T_2_ APP	7.15 (0.96)	7.88 (1.33)	−0.73	1.54	−0.47 (234.85)	.64	≥.99
T_2_ COS vs T_2_ Waitlist	7.15 (0.96)	7.52 (1.23)	−0.37	1.45	−0.25 (234.39)	.80	≥.99
T_2_ APP vs T_2_ Waitlist	7.88 (1.33)	7.52 (1.23)	0.36	1.70	0.21 (232.73)	.83	≥.99
T_3_ COS vs T_3_ APP	8.22 (0.98)	6.45 (1.41)	1.77	1.62	1.1 (270.36)	.27	≥.99
T_3_ COS vs T_3_ Waitlist	8.22 (0.98)	7.68 (1.32)	0.54	1.54	0.35 (278.59)	.72	≥.99
T_3_ APP vs T_3_ Waitlist	6.45 (1.41)	7.68 (1.32)	−1.23	1.82	−0.67 (286.75)	.50	≥.99

a The two EMM columns represent the first and second conditions being compared in each row, respectively. EMM: estimated marginal mean.

b All *t* tests were 2-tailed.

c *P*_holm_: Holm-Bonferroni corrected *P* values.

d BDI-II: Beck Depression Inventory-II.

e COS: cognitive behavioral therapy–based online self-help group.

f APP: cognitive behavioral therapy–based mobile application group.

g Waitlist: waitlist control group.

h BSS: Beck Scale for Suicide Ideation.

**Table 4.** Mean (SD) and effect sizes for primary and secondary outcomes.

Outcome, time points, and group	Mean (SD)	Effect size (Cohen *d*)	
		Within-group	Between-group
BDI-II^a^			
T_1_			
COS^b^	27.03 (10.01)	—^c^	—
APP^d^	26.72 (11.31)	—	—
Waitlist^e^	26.48 (9.93)	—	—
T_2_			
COS	14.57 (10.99)	1.38	—
APP	16.35 (10.87)	0.93	COS vs APP: 0.16
Waitlist	21.87 (10.42)	0.64	COS vs Waitlist: 0.68
T_3_			
COS	15.82 (12.86)	1.12^f^	—
APP	12.03 (10.20)	1.35^f^	COS vs APP: −0.33
Waitlist	21.53 (11.46)	0.48^f^	COS vs Waitlist: 0.47
BSS^g^			
T_1_			
COS	9.78 (7.11)	—	—
APP	8.31 (8.36)	—	—
Waitlist	7.54 (7.97)	—	—
T_2_			
COS	6.01 (7.08)	0.71	—
APP	7.08 (8.94)	0.23	COS vs APP: 0.13
Waitlist	6.54 (7.71)	025	COS vs Waitlist: 0.07
T_3_			
COS	7.40 (8.74)	0.33^f^	—
APP	4.61 (6.04)	0.59^f^	COS vs APP: –0.37
Waitlist	7.68 (8.07)	−0.02^f^	COS vs Waitlist: 0.03

a BDI-II: Beck Depression Inventory-II.

b COS: cognitive behavioral therapy–based online self-help group.

c Not applicable.

d APP: cognitive behavioral therapy–based mobile application group.

e Waitlist: waitlist control group.

f Within-group effect sizes for T_3_ were calculated relative to the baseline (T_1_) score.

g BSS: Beck Scale for Suicide Ideation.

### RCI Analysis

The RCI analysis included participants who completed both the T_1_ and T_2_ assessments. In the COS group, 76 participants completed the T_2_ assessment; however, one individual did not complete the baseline assessment and was therefore excluded, resulting in a sample of 75 participants. The APP and waitlist control groups had 37 and 46 participants, respectively. In the COS group, 75% (56/75) showed a significant change from pre- to postinterventions. About 57% (21/37) of participants in the APP group and 35% (16/46) in the waitlist control group demonstrated significant changes over the same period. The difference in the proportion of participants with reliable change between the COS and APP groups was marginally significant (*P*=.054).

### Secondary Outcomes

Within-subject dependence in suicidal ideation was identified (ICC=0.72), supporting the use of LMM. For suicidal ideation (as measured by the BSS), the LMM analysis revealed a significant main effect of time (*P*<.001) and a trend toward a time × group interaction (*P*=.09). The main effect of the group was not statistically significant (*P*=.80).

Figure 3B presents changes in suicidal ideation over time (T_1_ – T_3_) by group. Post hoc within-group comparisons indicated a reduction in suicidal ideation from T_1_ to T_2_ in the COS group (difference=3.13; *t*_277.11_=4.55, two-tailed; *P*<.001, *P*_holm_<.001; *d*=0.71), with no significant change between T_2_ and T_3_ (difference=−1.07; *t*_274.21_=−1.5, two-tailed; *P*=.13). In the APP group, the change from T_1_ to T_2_ was not statistically significant (difference=1.2; *t*_272.63_=1.27, two-tailed; *P*=.21). The comparison between T_1_ and T_3_ indicated a reduction, but did not reach the threshold for significance after Holm-Bonferroni correction (difference=2.63; *t*_278.73_=2.53, two-tailed; *P*=.01, *P*_holm_=.44). As secondary evidence, the waitlist control group showed minimal change in suicidal ideation over time. Neither the comparison between T_1_ and T_2_ (difference=0.69; *t*_273.74_=0.80, two-tailed; *P*=.43) nor the comparison between T_2_ and T_3_ (difference=−0.16; *t*_276.51_=−0.16, two-tailed; *P*=.87) reached statistical significance.

### Exploratory Outcomes

Intraclass correlations for the exploratory outcomes also demonstrated notable within-participant dependence, with ICCs ranging from 0.48 to 0.73.

There were significant main effects of time and time × group interaction for all exploratory outcomes (all *P*<.05), with the exception of perceived available support (as measured by PAS) (*P*=.75) (Multimedia Appendix 1).

At postintervention, both the COS and APP groups showed significant improvements in multiple domains: reduced perceived stress (PSS), dysfunctional attitudes (DAS-17), and loneliness (UCLA-LS), as well as increased self-efficacy (GSES), perceived social support (PAS), and reasons for living (RFL-YA) (all *P*<.05). In contrast, the waitlist control group showed no significant change across any of these outcomes between T_1_ and T_2_ (all *P*>.05).

Most improvements observed at T_2_ were sustained at the 3-month follow-up, with no significant changes from T_2_ for any outcomes except DAS-17 and UCLA-LS in the COS group. Specifically, DAS-17 scores increased (*P*=.02), and loneliness scores decreased (*P*=.04) from T_2_ to T_3_; however, neither result remained significant after applying the Holm-Bonferroni correction (*P*_holm_=.56 and *P*_holm_=.98, respectively).

At T_3_, the APP group showed higher GSES and RFL-YA scores than the waitlist control group (*P*=.006 and *P*=.03, respectively); however, these differences did not reach statistical significance after correction (*P*_holm_=.16 and *P*_holm_=.95, respectively). No significant between-group differences were observed for other variables at follow-up.

## Discussion

### Principal Findings

The primary objective of this RCT study was to evaluate the efficacy of a CBT-based online self-help group program (COS) for individuals experiencing depressive symptoms. Findings indicated that participants in the COS group showed a significant reduction in both depressive symptoms and suicide ideation, with these effects largely maintained at the 3-month follow-up. Notably, 75% (56/75) of those in the COS group exhibited clinically meaningful improvement in depressive symptoms, as determined by the RCI. At baseline, depressive symptoms in the COS group were of moderate severity, but following the intervention, their average symptom levels fell within the mild range. This level of improvement is comparable to outcomes typically achieved through standard, therapist-delivered CBT [57].

Although we expected the COS program to be at least as efficacious as the APP intervention for general CBT-based outcomes and potentially superior for outcomes influenced by peer interaction, the results revealed that the app-based program yielded similar improvement in reducing depressive symptoms, perceived stress, dysfunctional attitudes, and loneliness. This parity in outcomes is likely attributable to the shared therapeutic components across the COS and APP conditions. In both programs, participants engaged in cognitive restructuring exercises, including the identification of cognitive distortions within hypothetical scenarios and the generation of more adaptive, alternative thoughts. These fundamental CBT techniques were consistently applied in both conditions and may have contributed to the comparable levels of improvement observed.

Beyond the shared therapeutic elements, several intervention-related features may help explain why COS and APP yielded largely comparable outcomes. The interpersonal features unique to COS, such as validation, reciprocal feedback, and collaborative cognitive restructuring, may require a longer intervention period or greater group contact for their unique benefits to emerge fully. In this study, the COS program consisted of 7 sessions delivered over 3 weeks, which is considerably shorter than most peer support or group-based depression interventions that typically span 10 to 12 sessions [36]. The relatively brief structure may have limited the development of group processes that could otherwise differentiate COS from APP.

Implementation fidelity is another relevant consideration [58]. The APP condition delivered identical content with consistent pacing for every participant, ensuring high consistency. Conversely, the COS format, although manualized, naturally introduced variability in discussion depth, interaction patterns, and pacing across groups. While this variability may have supported peer engagement, it may also reduce the consistency with which CBT skills were practiced compared to the standardized app.

Despite the similarity in outcomes, the COS group demonstrated a marginally higher proportion of participants achieving clinically significant change, as indicated by the RCI, compared to the APP group. Furthermore, the analysis for suicide ideation revealed a trend-level time × condition interaction, with a significant reduction emerging only within the COS group. Post hoc comparisons indicated a statistically significant decrease in suicidal ideation from T_1_ to T_2_ only in the COS condition, even though the intervention did not explicitly target suicidality; in contrast, no significant within-group changes were detected in the APP or waitlist control groups. This finding is particularly salient, given the elevated suicide risk commonly associated with depressive disorders [59,60]. The reduction in suicidal ideation may be attributable to the relational dynamics of the self-help group format. One possible explanation is the supportive and interactive nature of the group setting, which may have provided emotional validation, normalization, and interpersonal connection—factors that have been identified as protective in the context of suicide risk [61]. Previous research has demonstrated that group-based interventions can be effective in reducing suicidal thoughts, with social support and a sense of belonging identified as key mechanisms [62,63]. Recently, peer-led interventions have gained increasing attention in suicide prevention efforts and have been recommended for their potential to meaningfully integrate lived experience into mental health care [64]. However, their adoption in practice remains limited. The COS program may offer a scalable and accessible intervention that not only aligns with these emerging recommendations but also addresses the current gaps in service delivery.

The COS program was specifically designed to integrate structured CBT techniques within a peer-led, self-help group framework. This hybrid model aimed to combine the clinical efficacy of CBT with the emotional resonance and empowerment afforded by lived-experience sharing, which is characteristic of self-help groups [65]. Importantly, since this low-intensity psychological intervention was manualized, brief in duration, and minimally dependent on professional facilitation [66], these features render the COS program particularly amenable to digital dissemination, offering a scalable and resource-efficient option for addressing the treatment gap in depression care.

### Limitations

This study has several limitations that must be acknowledged. First, although the COS program was designed for peer-led delivery, trained facilitators with psychological knowledge were present during sessions to ensure participant safety and adherence to the intervention protocol. While this approach helped maintain fidelity and manage potential risks, it may have reduced the ecological validity of a fully peer-led mode. We envision that future implementation will use a stepped-care approach, in which professionals initially lead the groups before transitioning leadership to peers. Consequently, future studies should evaluate the comparative efficacy of the COS program when delivered exclusively by peers versus professionals. This will help establish the viability of this scalable model and its clinical effectiveness in real-world settings. Second, the waitlist control group was recruited independently from the randomized intervention groups. Although efforts were made to match key sociodemographic characteristics, the absence of full randomization may have introduced unmeasured confounding variables, including differences in participants’ motivation or expectations, as well as potential time-related confounds that limit the strength of causal inferences. Third, all outcomes were measured using self-report instruments. Although standardized instruments with strong psychometric support were used, the exclusive reliance on self-report measures may introduce reporting bias. Future studies may benefit from incorporating clinician-administered assessments or objective indicators, such as behavioral or physiological measures. Fourth, blinding procedures could not be implemented due to the behavioral nature of the intervention and its peer-supported, online-delivery format. Although outcome assessments were completed directly by participants, thereby limiting the risk of assessor-rated bias, the absence of participant blinding remains a methodological limitation that should be considered when interpreting the results. Fifth, the participants were predominantly female and consisted largely of young adults, with an average age in the late twenties. This consideration is particularly pertinent for outcomes such as suicidal ideation, where gender differences in prevalence and expression are well documented. Although the inclusion criteria permitted participation up to age 55 years, the reliance on online recruitment methods likely contributed to this skew. Consequently, this demographic profile may limit the generalizability of the findings to older adults or populations with different digital usage patterns. Finally, the study did not include long-term follow-up beyond 3 months. Future studies should assess whether the observed improvements are sustained over extended periods, including follow-ups of at least 12 months, and further evaluate the real-world applicability of fully peer-led implementations.

### Conclusions

This study contributes valuable evidence supporting digital approaches to depression and suicidality. The continued use of remote, group-based psychological interventions may help address persistent barriers to accessing conventional services. Within this context, the development and evaluation of scalable, evidence-based digital programs remain an important public health objective, particularly for populations with limited access to mental health professionals. Notably, the findings highlight the potential of structured self-help group interventions that can be delivered with minimal professional involvement, offering a practical and accessible option for expanding psychological support.

## Supplementary material

10.2196/76028 Multimedia Appendix 1 Exploratory outcome measures.

10.2196/76028 Checklist 1 CONSORT-eHEALTH (V1.6.1) checklist.

## Abbreviations

APP: cognitive behavioral therapy–based mobile application group
BDI-II: Beck Depression Inventory-II
BSS: Beck Scale for Suicide Ideation
BSSS: Berlin Social Support Scale
CBT: cognitive behavioral therapy
CONSORT: Consolidated Standards of Reporting Trials
COS: cognitive behavioral therapy–based online self-help group
DAS-17: Dysfunctional Attitude Scale (Form A) Revised
DSM-5: Diagnostic and Statistical Manual of Mental Disorders, 5th Edition
GSES: General Self-Efficacy Scale
ICC: intraclass correlation coefficient
LMM: linear mixed model
MAR: missing at random
PAS: Perceived Available Support
PHQ-9: Patient Health Questionnaire-9
PSS: Perceived Stress Scale
RCI: reliable change index
RCT: randomized controlled trial
RFL-YA: Reasons for Living Inventory for Young Adults
SCID-5-CV: Structured Clinical Interview for DSM-5 – Clinician Version
UCLA: University of California, Los Angeles
UCLA-LS: UCLA Loneliness Scale version 3
