# Supplementary material for: Efficacy of a Cognitive Behavioral Therapy–Based Online Self-Help Group for Depression and Suicide Ideation: Randomized Controlled Trial
Source: JMIR Mhealth Uhealth. 2026 Jul 3;14:e76028. doi: 10.2196/76028 (PMC13331252; doi:10.2196/76028)
Supplement: Multimedia Appendix 1 [file mhealth-v14-e76028-s001.docx]

**Table S1.** Estimated marginal means and standard deviations of exploratory outcome measures across time.

|  |  | T_1_ (Baseline) | | | | T_2_ (Post) | | | | T_3_ (3-month follow-up) | | | |
| --- | --- | --- | --- | --- | --- | --- | --- | --- | --- | --- | --- | --- | --- |
|  |  |  |  | 95% CI | |  |  | 95% CI | |  |  | 95% CI | |
|  |  | EMM^*^ | SE | Lower | Upper | EMM^*^ | SE | Lower | Upper | EMM^*^ | SE | Lower | Upper |
| DAS | COS | 70.89 | 2.05 | 66.85 | 74.93 | 60.04 | 2.08 | 55.94 | 64.14 | 63.76 | 2.14 | 59.55 | 67.97 |
|  | APP | 67.89 | 2.87 | 62.24 | 73.53 | 58.66 | 2.9 | 52.95 | 64.38 | 56.8 | 3.06 | 50.77 | 62.83 |
|  | Waitlist | 67.07 | 2.66 | 61.84 | 72.31 | 66.23 | 2.68 | 60.95 | 71.51 | 68.05 | 2.86 | 62.41 | 73.68 |
| PSS | COS | 24.2 | 0.68 | 22.86 | 25.54 | 19.81 | 0.7 | 18.44 | 21.18 | 20.96 | 0.72 | 19.54 | 22.39 |
|  | APP | 24.76 | 0.95 | 22.88 | 26.63 | 20.91 | 0.97 | 19 | 22.82 | 19.6 | 1.05 | 17.53 | 21.68 |
|  | Waitlist | 25.04 | 0.88 | 23.3 | 26.77 | 23.39 | 0.9 | 21.63 | 25.16 | 21.93 | 0.99 | 19.98 | 23.87 |
| SE | COS | 61.36 | 1.61 | 58.19 | 64.53 | 68.78 | 1.63 | 65.57 | 71.99 | 67.95 | 1.67 | 64.66 | 71.23 |
|  | APP | 63.55 | 2.25 | 59.12 | 67.98 | 69.32 | 2.27 | 64.85 | 73.8 | 72.09 | 2.38 | 67.4 | 76.79 |
|  | Waitlist | 62.84 | 2.08 | 58.74 | 66.95 | 64.73 | 2.1 | 60.59 | 68.87 | 63.48 | 2.23 | 59.1 | 67.86 |
| UCLA-LS | COS | 32.56 | 1.24 | 30.11 | 35 | 29.44 | 1.26 | 26.96 | 31.92 | 27.39 | 1.29 | 24.85 | 29.94 |
|  | APP | 31.66 | 1.74 | 28.24 | 35.08 | 25.37 | 1.76 | 21.91 | 28.83 | 24.65 | 1.85 | 21 | 28.3 |
|  | Waitlist | 29.01 | 1.61 | 25.83 | 32.18 | 27.49 | 1.62 | 24.29 | 30.69 | 27.8 | 1.73 | 24.39 | 31.21 |
| PAS | COS | 22.38 | 0.55 | 21.3 | 23.46 | 24.01 | 0.56 | 22.92 | 25.11 | 23.61 | 0.58 | 22.48 | 24.75 |
|  | APP | 22.79 | 0.76 | 21.29 | 24.29 | 25.05 | 0.77 | 23.53 | 26.57 | 24.41 | 0.83 | 22.78 | 26.05 |
|  | Waitlist | 23.13 | 0.71 | 21.75 | 24.52 | 24.12 | 0.71 | 22.72 | 25.53 | 23.92 | 0.78 | 22.39 | 25.45 |
| RFL-YA | COS | 3.2 | 0.11 | 2.98 | 3.42 | 3.59 | 0.11 | 3.36 | 3.81 | 3.68 | 0.12 | 3.45 | 3.91 |
|  | APP | 3.4 | 0.16 | 3.1 | 3.71 | 3.71 | 0.16 | 3.41 | 4.02 | 3.88 | 0.17 | 3.55 | 4.2 |
|  | Waitlist | 3.38 | 0.14 | 3.1 | 3.67 | 3.36 | 0.15 | 3.07 | 3.65 | 3.42 | 0.15 | 3.11 | 3.72 |

**Table S2.** Post-hoc Time × Group comparison for the exploratory outcome measures.

|  | Comparison | | | | |  | | | | | |
| --- | --- | --- | --- | --- | --- | --- | --- | --- | --- | --- | --- |
| Outcome | Time | Group |  | Time | Group | Difference | SE | *t* | df | *P* | *P*_holm_ |
| DAS-17 | T_1_ | COS | - | T_2_ | COS | 10.85 | 1.56 | 6.95 | 284.36 | < .001 | < .001 |
|  | T_1_ | APP | - | T_2_ | APP | 9.22 | 2.21 | 4.18 | 282.18 | < .001 | .001 |
|  | T_1_ | Waitlsist | - | T_2_ | Waitlist | 0.85 | 2.01 | 0.42 | 283.36 | .68 | 1 |
|  | T_2_ | COS | - | T_3_ | COS | -3.72 | 1.63 | -2.28 | 282.24 | .023 | .56 |
|  | T_2_ | APP | - | T_3_ | APP | 1.86 | 2.43 | 0.77 | 285.92 | .44 | 1 |
|  | T_2_ | Waitlist | - | T_3_ | Waitlist | -1.82 | 2.25 | -0.81 | 285.61 | .42 | 1 |
|  | T_2_ | COS | - | T_2_ | APP | 1.38 | 3.36 | 0.41 | 248.72 | .68 | 1 |
|  | T_2_ | COS | - | T_2_ | Waitlist | -6.19 | 3.17 | -1.95 | 248 | .052 | 1 |
|  | T_2_ | APP | - | T_2_ | Waitlist | -7.57 | 3.71 | -2.04 | 248.09 | .043 | .89 |
|  | T_3_ | COS | - | T_3_ | APP | 6.96 | 3.54 | 1.97 | 285.44 | .05 | 1 |
|  | T_3_ | COS | - | T_3_ | Waitlist | -4.29 | 3.36 | -1.28 | 291.77 | .20 | 1 |
|  | T_3_ | APP | - | T_3_ | Waitlist | -11.25 | 3.98 | -2.83 | 299.27 | .005 | .15 |
| PSS | T_1_ | COS | - | T_2_ | COS | 4.39 | 0.67 | 6.59 | 287.13 | < .001 | < .001 |
|  | T_1_ | APP | - | T_2_ | APP | 3.84 | 0.94 | 4.08 | 284.47 | < .001 | .002 |
|  | T_1_ | Waitlsist | - | T_2_ | Waitlist | 1.64 | 0.86 | 1.91 | 285.74 | .057 | .973 |
|  | T_2_ | COS | - | T_3_ | COS | -1.15 | 0.7 | -1.65 | 285.65 | .10 | 1 |
|  | T_2_ | APP | - | T_3_ | APP | 1.31 | 1.04 | 1.26 | 291.69 | .21 | 1 |
|  | T_2_ | Waitlist | - | T_3_ | Waitlist | 1.47 | 0.96 | 1.53 | 292.18 | .13 | 1 |
|  | T_2_ | COS | - | T_2_ | APP | -1.1 | 1.14 | -0.97 | 317.95 | .33 | 1 |
|  | T_2_ | COS | - | T_2_ | Waitlist | -3.58 | 1.07 | -3.35 | 317.18 | < .001 | .024 |
|  | T_2_ | APP | - | T_2_ | Waitlist | -2.48 | 1.25 | -1.98 | 317.75 | .048 | .89 |
|  | T_3_ | COS | - | T_3_ | APP | 1.36 | 1.22 | 1.11 | 361.28 | .27 | 1 |
|  | T_3_ | COS | - | T_3_ | Waitlist | -0.96 | 1.17 | -0.82 | 368.62 | .41 | 1 |
|  | T_3_ | APP | - | T_3_ | Waitlist | -2.32 | 1.39 | -1.67 | 375.46 | .095 | 1 |
| GSES | T_1_ | COS | - | T_2_ | COS | -7.42 | 1.14 | -6.52 | 283.67 | < .001 | < .001 |
|  | T_1_ | APP | - | T_2_ | APP | -5.77 | 1.61 | -3.58 | 281.67 | < .001 | .013 |
|  | T_1_ | Waitlsist | - | T_2_ | Waitlist | -1.89 | 1.47 | -1.29 | 282.78 | .19 | 1 |
|  | T_2_ | COS | - | T_3_ | COS | 0.83 | 1.19 | 0.7 | 281.62 | .49 | 1 |
|  | T_2_ | APP | - | T_3_ | APP | -2.77 | 1.77 | -1.56 | 284.81 | .12 | 1 |
|  | T_2_ | Waitlist | - | T_3_ | Waitlist | 1.25 | 1.64 | 0.76 | 284.42 | .45 | 1 |
|  | T_2_ | COS | - | T_2_ | APP | -0.54 | 2.63 | -0.21 | 235.4 | .84 | 1 |
|  | T_2_ | COS | - | T_2_ | Waitlist | 4.05 | 2.47 | 1.64 | 234.73 | .10 | 1 |
|  | T_2_ | APP | - | T_2_ | Waitlist | 4.59 | 2.9 | 1.58 | 234.75 | .12 | 1 |
|  | T_3_ | COS | - | T_3_ | APP | -4.15 | 2.75 | -1.51 | 268.2 | .13 | 1 |
|  | T_3_ | COS | - | T_3_ | Waitlist | 4.47 | 2.61 | 1.71 | 273.83 | .09 | 1 |
|  | T_3_ | APP | - | T_3_ | Waitlist | 8.61 | 3.08 | 2.79 | 280.79 | .006 | .16 |
| UCLA-LS | T_1_ | COS | - | T_2_ | COS | 3.12 | 0.93 | 3.35 | 284.19 | < .001 | .028 |
|  | T_1_ | APP | - | T_2_ | APP | 6.29 | 1.31 | 4.79 | 282.05 | < .001 | < .001 |
|  | T_1_ | Waitlsist | - | T_2_ | Waitlist | 1.52 | 1.2 | 1.27 | 283.22 | .21 | 1 |
|  | T_2_ | COS | - | T_3_ | COS | 2.05 | 0.97 | 2.1 | 282.09 | .036 | .98 |
|  | T_2_ | APP | - | T_3_ | APP | 0.72 | 1.45 | 0.5 | 285.64 | .62 | 1 |
|  | T_2_ | Waitlist | - | T_3_ | Waitlist | -0.31 | 1.34 | -0.23 | 285.31 | .82 | 1 |
|  | T_2_ | COS | - | T_2_ | APP | 4.07 | 2.04 | 2 | 245.4 | .047 | 1 |
|  | T_2_ | COS | - | T_2_ | Waitlist | 1.95 | 1.92 | 1.02 | 244.69 | .31 | 1 |
|  | T_2_ | APP | - | T_2_ | Waitlist | -2.12 | 2.25 | -0.94 | 244.76 | .35 | 1 |
|  | T_3_ | COS | - | T_3_ | APP | 2.75 | 2.14 | 1.28 | 281.21 | .20 | 1 |
|  | T_3_ | COS | - | T_3_ | Waitlist | -0.4 | 2.03 | -0.2 | 287.38 | .84 | 1 |
|  | T_3_ | APP | - | T_3_ | Waitlist | -3.15 | 2.4 | -1.31 | 294.77 | .19 | 1 |
| PAS | T_1_ | COS | - | T_2_ | COS | -1.63 | 0.49 | -3.31 | 285.78 | .001 | .038 |
|  | T_1_ | APP | - | T_2_ | APP | -2.26 | 0.69 | -3.29 | 281.58 | .001 | .04 |
|  | T_1_ | Waitlsist | - | T_2_ | Waitlist | -0.99 | 0.63 | -1.58 | 282.86 | .12 | 1 |
|  | T_2_ | COS | - | T_3_ | COS | 0.4 | 0.51 | 0.78 | 282.42 | .44 | 1 |
|  | T_2_ | APP | - | T_3_ | APP | 0.64 | 0.76 | 0.84 | 287.2 | .40 | 1 |
|  | T_2_ | Waitlist | - | T_3_ | Waitlist | 0.2 | 0.7 | 0.29 | 287.27 | .77 | 1 |
|  | T_2_ | COS | - | T_2_ | APP | -1.04 | 0.9 | -1.15 | 286.5 | .25 | 1 |
|  | T_2_ | COS | - | T_2_ | Waitlist | -0.11 | 0.85 | -0.13 | 285.76 | .89 | 1 |
|  | T_2_ | APP | - | T_2_ | Waitlist | 0.93 | 0.99 | 0.93 | 286.03 | .35 | 1 |
|  | T_3_ | COS | - | T_3_ | APP | -0.8 | 0.96 | -0.83 | 328.63 | .41 | 1 |
|  | T_3_ | COS | - | T_3_ | Waitlist | -0.31 | 0.92 | -0.34 | 335.93 | .74 | 1 |
|  | T_3_ | APP | - | T_3_ | Waitlist | 0.49 | 1.09 | 0.45 | 344.72 | .65 | 1 |
| RFL-YA | T_1_ | COS | - | T_2_ | COS | -0.39 | 0.08 | -4.66 | 282.36 | < .001 | < .001 |
|  | T_1_ | APP | - | T_2_ | APP | -0.31 | 0.12 | -2.71 | 278.92 | .007 | 0.224 |
|  | T_1_ | Waitlsist | - | T_2_ | Waitlist | 0.02 | 0.11 | 0.22 | 280.08 | .82 | 1 |
|  | T_2_ | COS | - | T_3_ | COS | -0.1 | 0.09 | -1.12 | 279.65 | .27 | 1 |
|  | T_2_ | APP | - | T_3_ | APP | -0.16 | 0.13 | -1.26 | 282.35 | .21 | 1 |
|  | T_2_ | Waitlist | - | T_3_ | Waitlist | -0.06 | 0.12 | -0.48 | 281.99 | .63 | 1 |
|  | T_2_ | COS | - | T_2_ | APP | -0.13 | 0.18 | -0.71 | 240.33 | .48 | 1 |
|  | T_2_ | COS | - | T_2_ | Waitlist | 0.23 | 0.17 | 1.31 | 239.67 | .19 | 1 |
|  | T_2_ | APP | - | T_2_ | Waitlist | 0.35 | 0.2 | 1.76 | 239.62 | .08 | 1 |
|  | T_3_ | COS | - | T_3_ | APP | -0.19 | 0.19 | -1.01 | 274.95 | .31 | 1 |
|  | T_3_ | COS | - | T_3_ | Waitlist | 0.26 | 0.18 | 1.46 | 280.82 | .15 | 1 |
|  | T_3_ | APP | - | T_3_ | Waitlist | 0.46 | 0.21 | 2.13 | 288.04 | .034 | .95 |
